# A Digital Lifestyle App for Hypertension During Pregnancy: Mixed Methods Intervention Development Study Using the Person-Based Approach

**DOI:** 10.2196/68927

**Published:** 2025-07-18

**Authors:** Lucy Goddard, Katherine Tucker, Nerys M Astbury, Cristian Roman, Yuan Chi, Katherine Morgan, Patricia Devitt, Richard J McManus

**Affiliations:** 1 Nuffield Department of Primary Care Health Sciences, University of Oxford, Radcliffe Observatory Quarter, Woodstock Road, Oxford, OX2 6GG, United Kingdom, 44 01865 ext 289215; 2 NIHR Oxford Biomedical Research Centre, University of Oxford, Oxford, United Kingdom; 3 Patient and Public Contributor, United Kingdom; 4 Brighton and Sussex Medical School, University of Brighton and University of Sussex, Brighton, United Kingdom

**Keywords:** pregnancy, hypertension, lifestyle, behavior change, person-based approach, intervention development, mobile phone

## Abstract

**Background:**

Chronic hypertension affects 1%‐5% of pregnancies, increasing women’s risks of adverse pregnancy outcomes and life-long cardiovascular disease risk. Therefore, care management during pregnancy includes close monitoring of blood pressure and medication. Healthy dietary and physical activity behaviors have proven beneficial effects on blood pressure outside and during pregnancy. However, little is known about the best way to support women with chronic hypertension during pregnancy to adopt such behaviors, which could improve pregnancy outcomes, as well as future cardiovascular health.

**Objective:**

This study aims to develop and optimize a digital lifestyle intervention—the DAPHNY (Diet and Activity for Pregnancy Hypertension) app—with those who have experienced chronic hypertension during pregnancy.

**Methods:**

Guided by the person-based approach to intervention development, a review of literature and continuous expert input, including from patient and public representatives, informed the planning stage. This was followed by focus groups with maternity health professionals (n=23) and think-aloud interviews with women who had experience of chronic hypertension during pregnancy (n=11). A content analysis, underpinned by theoretical modeling using the capability opportunity motivation-behavior model, informed 3 logic models to visualize modifications for meaningful engagement with an intervention and sustained behavior change. The intervention was modified iteratively, leading to a first version of the digital intervention that was tested by women (n=10) to further optimize acceptability and engagement. App use data and user engagement patterns were captured.

**Results:**

An evidence-based, theoretically informed lifestyle app, named DAPHNY, was developed. Key features included in logic models and implemented into a first version of the app comprised supportive messaging to acknowledge challenges of hypertensive pregnancy, goal setting and progress reports for feedback on behaviors, information about health consequences to shape knowledge, credible source endorsement, and a reward or recognition system to acknowledge effort had been made. Engagement with the DAPHNY app during user testing demonstrated variability across users, with a mean of 13 (SD 6.84) sessions per participant. Session duration was variable, with a median of 36 seconds (range: 5 seconds to 5 minutes, 20 seconds). Action-based pages, including recording blood pressure (40 sessions) and step count (39 sessions), were accessed more frequently than informational pages, which required a deeper level of app engagement.

**Conclusions:**

Development of the DAPHNY app, underpinned by an established behavioral framework for developing digital interventions, provided new data insights about how to support women with chronic hypertension to engage in healthy behaviors, a currently overlooked aspect of blood pressure management. Future iterations should focus on increasing engagement and supporting implementation through streamlined content and integration with existing health systems and self-monitoring data. Rigorous, larger-scale studies including comprehensive process evaluation would determine potential clinical effectiveness, implementation strategies, and impact for women and health care professionals.

## Introduction

Supporting women with pregnancies that are complicated by hypertension to adopt healthy behaviors could play an important role in blood pressure control during pregnancy and beyond [1]. However, an understanding of how best to support women with existing, chronic hypertension to manage their blood pressure and improve future cardiovascular health through targeted lifestyle modification during pregnancy is unknown [2,3].

Pregnant women with chronic hypertension are at increased risk of pregnancy and birth complications, including pre-eclampsia, which occurs in a quarter of women with chronic hypertension [4]. They have approximately double the risk of major adverse cardiovascular events in later life compared to women who do not have any hypertension during pregnancy [5]. The prevalence of chronic hypertension in pregnancy is increasing, likely due to rising maternal age and obesity rates in the population, which further increase pre-eclampsia risk [6-9]. This indicates a public health concern requiring attention [10].

Lifestyle behaviors, including diet and physical activity, play a vital role in preventing and managing cardiovascular disease and are well-evidenced to provide sustained, clinically relevant reductions in blood pressure, outside of pregnancy [1]. Weight loss is a key recommendation for hypertension management outside of pregnancy, but as weight loss is not recommended during pregnancy, attenuation of excessive gestational weight gain is the focus of lifestyle interventions to improve pregnancy and birth outcomes [11]. Syntheses of study-level data have produced mixed results, but there appears to be some, albeit low-quality, evidence that interventions may have a protective effect on the incidence of pre-eclampsia, hypertension, gestational diabetes, and preterm delivery [12-15]. A recent systematic review identified no studies that specifically developed and tested lifestyle interventions to support healthy lifestyle behaviors for women who have chronic hypertension, who may benefit the most from lifestyle modification [3]. Since this is an important population at risk of poor pregnancy experiences and outcomes, a clear gap in the literature exists, which this study aims to start addressing.

Delivering health promotion messaging in a way that has a meaningful impact on patient outcomes is a challenge within current National Health Service (NHS) models of care and system constraints. However, a shift toward prevention and proactive care, including the prevention of cardiovascular events, is an NHS priority [16]. Alongside this, improving digital systems to improve population health and support health professionals to deliver unbiased information is at the forefront of maternity service delivery, with its potential to increase women’s choice, women’s engagement with services, and facilitate existing care pathways [17].

App use in pregnancy is prolific, but many freely available apps lack robust scientific input, lack regulation of content, screening for comorbidities, health care professional involvement, and theoretical underpinning [18,19]. This is especially relevant for lifestyle information, which can be sought from alternative, potentially less reputable sources [20,21]. Encouragingly, remote monitoring apps to manage hypertensive pregnancy have been developed and robustly trialed on large populations and rolled out widely and found to be acceptable and feasible for women [22-25]. An intervention to manage chronic hypertension during pregnancy with embedded lifestyle support to meaningfully change behavior, that is collaboratively developed with women who have long-term hypertension, has not been scientifically explored and could be an important avenue to improving pregnancy, birth, and future cardiovascular health.

This intervention development study aimed to design, develop, and test a digital lifestyle intervention with women who have long-term hypertension and have been pregnant, and health care professionals.

This paper is reported following the GUIDED (Guidance for the Reporting of Intervention Development) checklist (Checklist 1) [26].

## Methods

### Design

**Figure 1.**
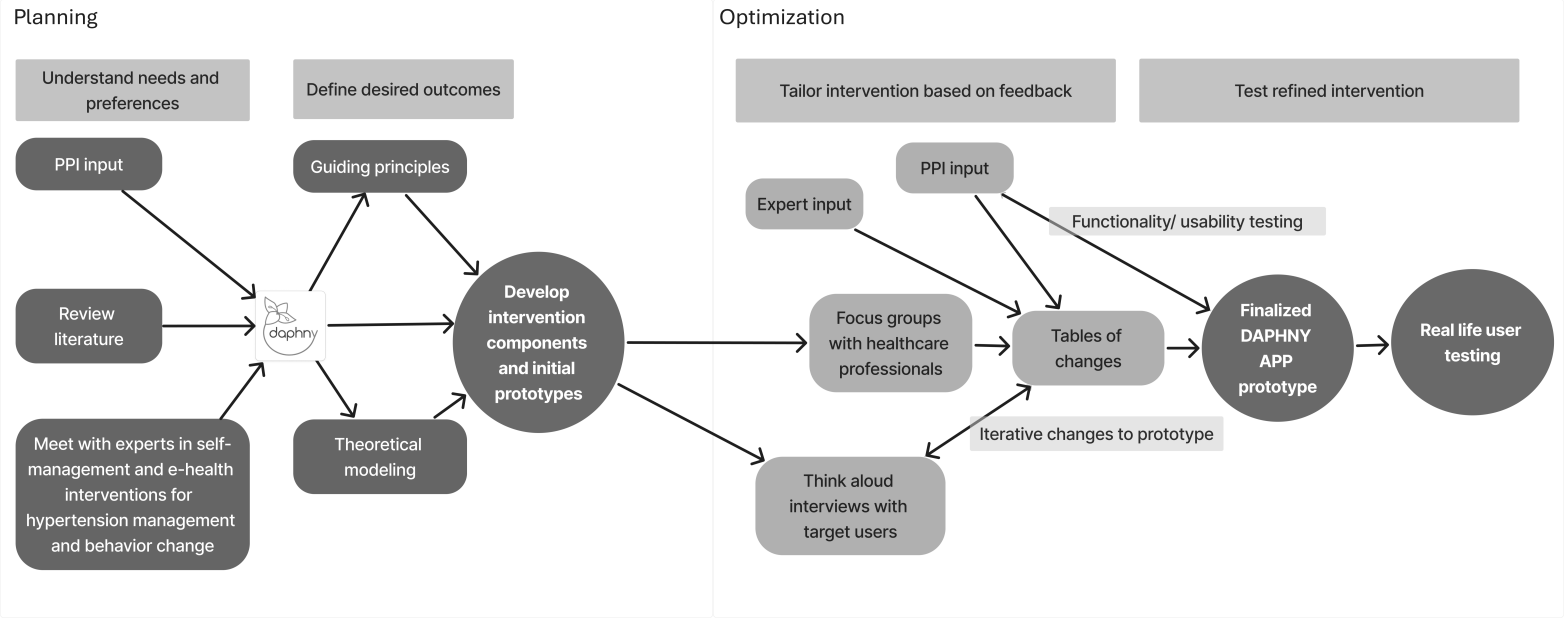
Planning and optimization phases of the person-based approach to develop the DAPHNY (Diet and Activity for Pregnancy Hypertension) intervention. PPI: patient and public involvement.

This intervention development study incorporated two phases of the person-based approach (PBA): (1) planning phase and (2) optimization phase. The PBA is frequently used in the development of digital interventions and incorporates user design methods alongside evidence-based behavior change methods, aiming to ensure that interventions address the needs of the target population, increasing their likelihood of successful roll-out [27,28]. There was fluidity between the two phases. A visual demonstration of this dynamic approach is displayed in Figure 1.

### Ethical Considerations

Ethical approval was gained from Health and Care Research Wales (REC ref: 22/WA/0130), and written informed consent was obtained from all participants. Participant data were deidentified as soon as it was practical to do so through assigning unique participant IDs. All women who took part in the study received a £10 (US $13.77) shopping voucher to thank them for their time and participation.

### Planning Phase

#### Patient and Public Involvement

Patient and public involvement (PPI) is vital to promote the interests of patients throughout the research cycle to shape more acceptable and sustainable study methodologies and interventions [29-31]. A group of 12 women with experience of hypertension during pregnancy met in groups or individually, providing an informal space to discuss current management of hypertension and ideas around what would support healthy behaviors during pregnancy, if this was something they were seeking to do. Recurring topics of discussion were that lifestyle advice was rarely discussed in relation to blood pressure, with some unaware that changes in diet and physical activity could reduce blood pressure. The potential to reduce medication if blood pressure improved was a motivating factor to engage in healthy behaviors for some, due to side effects acting as a barrier to being active, and some shared concerns around the impact on their baby.

Two PPI representatives joined the study team and had continuous involvement throughout to contribute ideas, support the study design and procedures, review study materials, and share perspectives on the developing intervention. This valuable insight contributed to optimizing the conduct of the study and the developed intervention.

#### Scoping the Literature

Exploring the literature was a necessarily rapid process. An overview of the foundation of evidence on which the study was built is summarized in Multimedia Appendix 1. This also serves to provide a rationale for the app prototypes that were further refined and iteratively developed.

#### Intervention Guiding Principles

Guiding principles summarize the intervention objectives [28]. Three key design objectives and the ways the intervention could address these were referred to throughout when making development decisions (Textbox 1).

Textbox 1. **Planning phase of the person-based approach—DAPHNY (Diet and Activity for Pregnancy Hypertension) intervention guiding principles.**

**Design objective 1: To increase awareness of the association between lifestyle and high blood pressure**

- Provide evidence for the association between diet and activity patterns and blood pressure

- Provide education on nutrients that support good blood pressure management

**Design objective 2: To motivate women and increase confidence to undertake positive and sustainable lifestyle changes during pregnancy**

- Emphasis on how to make small changes to build over time as a way to protect their own health in the short- and long-term health and a way to support their baby’s growth and development
- Create a platform that provides feedback to increase confidence and motivation
- Motivational messages to prompt movement
- Notifications to prompt uptake of healthy dietary habits
- Repetition of information to enable understanding
- Opportunity to self-monitor
- Acknowledgment of the barriers to change and ways to overcome these

**Design objective 3: Intervention content is simple, clear, and appropriate for lower health literacy**

- Short sentences, avoiding complex language, terminology, abbreviations, and jargon
- Create a platform that is visual, attractive, and easy to navigate
- Wording and content of the platform supportive and encouraging
- Content and messages piloted with a diverse group of women, including those whose first language is not English

#### Theoretical Modeling

**Figure 2.**
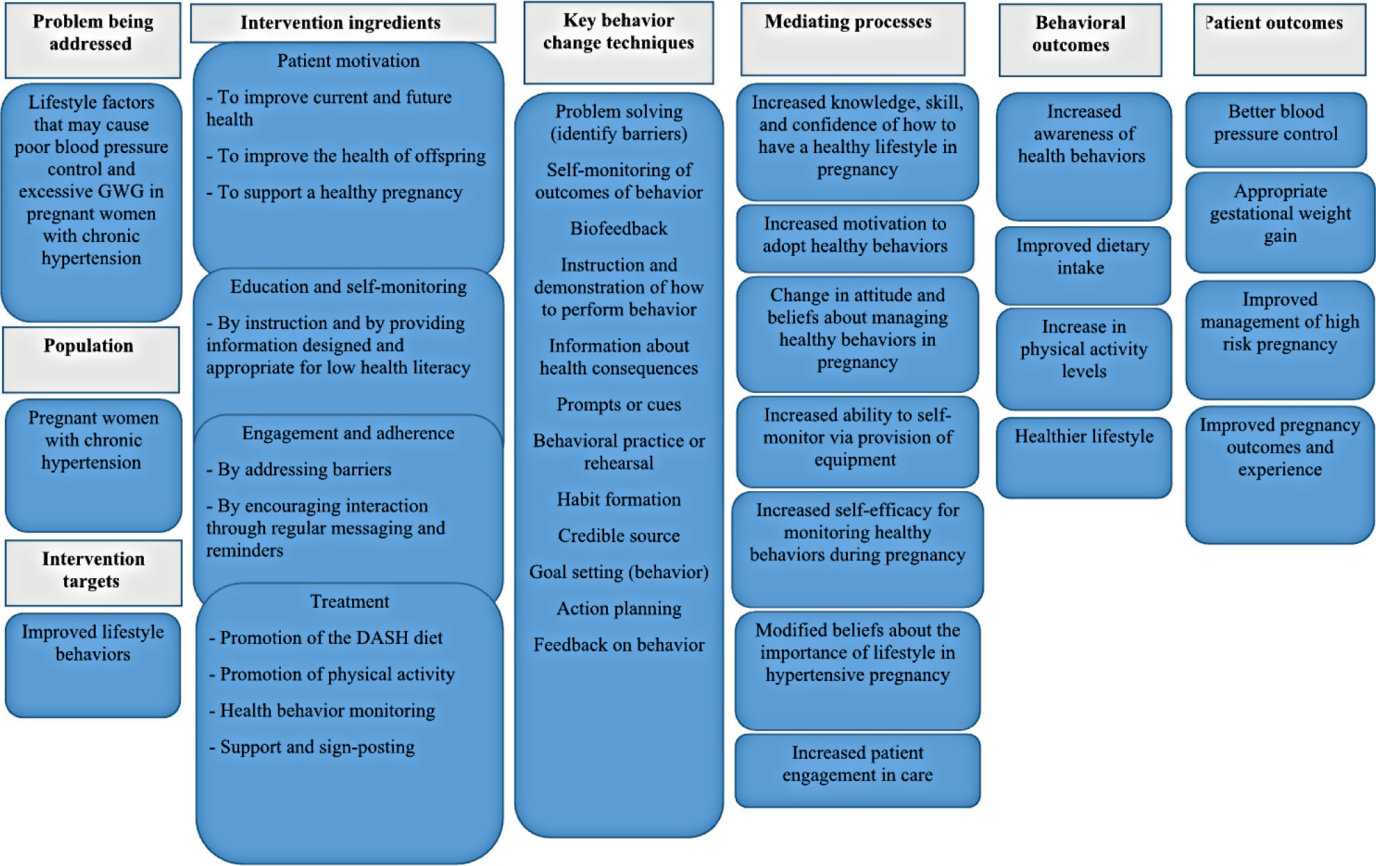
Planning phase of the person-based approach—introductory logic model of the digital lifestyle intervention. DASH: Dietary Approaches to Stop Hypertension; GWG: gestational weight gain.

The capability opportunity motivation-behavior model (COM-B model) underpinned the intervention development, given its incorporation of other theories and practical application through identifying key barriers and facilitators that disable or enable positive behavior change [32,33]. It posits that behavior change is the result of direct or indirect interactions between the components: capability (skills and knowledge), opportunity (available resources and environmental factors), and motivation (brain processes such as intentions, emotional responses, and beliefs) to enact a target behavior. An introductory logic model (Figure 2) hypothesized how the intervention may work. Logic models provide a visual and testable representation to hypothesize how an intervention and its features may work. These provide fine-grain detail on the barriers, facilitators, and implemented features and behavior change techniques (BCTs), the smallest components of an intervention that influence behavior, to address these [34].

### Optimization Phase

#### Overview

Focus groups and early feasibility testing involving think-aloud interviews and user testing took place at the maternity units within Oxford University Hospital Trust and Manchester University Foundation Trust. All focus groups and interviews were conducted by the lead researcher.

#### Health Care Professional Focus Groups

Focus groups with maternity health care professionals were carried out to understand views of the developing intervention, as well as important insight into experiences of hypertension management and lifestyle advice for this population.

##### Recruitment

Participants were any health care professional providing care for hypertensive pregnancies with varying experience working in different clinical settings. Participant information was shared, and time was given to consider participation prior to obtaining informed consent and commencing study activities.

##### Data Collection

Semistructured interviews or focus groups involved discussions about usual care pathways for pregnant women with chronic hypertension and the experience of providing lifestyle advice. Prototypes of the app were presented, using FIGMA (a web app for user interface design), to stimulate discussion and questions oriented around these. Discussions were fluid between directly relating to the intervention, to wider challenges experienced, and views on providing care for these women.

### Early Feasibility Testing With Women

#### Overview

Early feasibility testing involved first, and in parallel to health care professional focus groups, think-aloud interviews to design and refine intervention features. Subsequently, user testing to understand the use of the developed intervention in real-life contexts was conducted.

#### Recruitment

Participants were pregnant, or recently pregnant (for think-aloud interviews), women with chronic hypertension. For the user testing, participants were required to be currently pregnant and have a compatible smartphone (Android or iOS) for the developed DAPHNY (Diet and Activity for Pregnancy Hypertension) app.

Purposive sampling was used to recruit eligible participants from specialist antenatal clinics, antenatal wards, or via email. Information about the study was provided, and following time to consider participation, consent was obtained prior to study enrollment.

#### Data Collection

Think-aloud interviews are an evidence-based qualitative interview technique commonly adopted in the PBA to gather important insights from target users about how the intervention can be adapted to meet their needs and overcome barriers [35,36].

Interviews began with general questions about the experience of lifestyle advice in the context of hypertension, as well as current behaviors (lifestyle or other) to manage their blood pressure. Participants then freely explored the prototype, navigating into different intervention components as they wished, sharing immediate reactions and thoughts throughout to establish user acceptability and inform changes. Prompts used were informed by the COM-B model, and questions asked responded to women’s interaction with the prototype to elicit the most useful information for intervention development and optimization.

Following think-aloud interviews, user testing with a different group of participants was carried out. Participants used the developed DAPHNY app at home within their daily lives for at least 2 weeks, and app use data were collected. At enrollment, participants were given access to the DAPHNY app. An initial orientation was given, and brief written instructions were provided. Qualitative interviews following this period were carried out and will be published elsewhere.

#### Qualitative Data Management

Data collection continued until no new major information was appearing with additional interviews, that is, comments related more to personal preferences than key intervention functions that influenced user engagement or behavior change, as recommended in the PBA.

All focus groups and interviews were audio-recorded, deidentified, and transcribed by the lead researcher or an independent transcriber. All quotes directly relating to the intervention design, content, component, and features were extracted and tabulated into an Excel (Microsoft Corp) workbook, which organized and managed the dataset for a content analysis.

### Analysis

#### Health Care Professional Focus Groups and Early Feasibility Testing, Think-Aloud Interviews

Content analysis was conducted throughout data collection so that modifications were made and then shown to subsequent participants. There were 3 main iterations throughout data collection with minor changes in between. Each comment was considered against a coding framework, commonly used in PBA, including important for behavior change, easy and uncontroversial, said repeatedly, supported by experience, does not contradict, and not changed, to support decision-making about potential intervention modification (Multimedia Appendix 2). The MoSCOW (Must have, Should have, Could have, Would like) prioritization model, commonly used for developing digital interventions, prioritized changes to make [37]. A “table of changes” documented the decisions and actions throughout the process, providing an audit trail of how the intervention developed over the course of the study.

The “table of changes” was summarized into logic models and data mapped against the COM-B model to define what participants felt the barriers and facilitators were to its use and engagement. Modifications to the intervention with the associated BCT, which were derived from the behavior change taxonomy, were detailed in the logic model [34], successfully linking the data to core elements of the behavior change wheel (COM-B) that underpins the intervention.

#### Early Feasibility Testing, User Testing

App use logbooks with raw data for each app page were exported in CSV format and analyzed in Excel. The following insights were obtained: the total duration of app use (including number of days and minutes), the duration and number of individual sessions (presented as mean, SD, median, and range), and the pages that were most frequently accessed. This data is presented in summary graphs and tables to provide a view of how the app was used. Data entered by the participants, including step count and blood pressure, are reported descriptively.

Data extracted included sessions occurring 20 minutes after initial installation of the app (to exclude the initial orientation session) to 5 PM on the date of the participant interview. A session was defined from the moment a participant opened the app until they exited the app, or if the app was not exited but it was running in the background, but the participant did not return to it again within half an hour. Sessions that were less than 5 seconds long were removed from the analysis to exclude any unintentional interactions that were too short to be meaningful [38]. Though this removed interactions such as looking at notifications, it meant that the higher-level pages did not appear as the most popular.

## Results

### Participant Characteristics

A multidisciplinary mix of 23 health care professionals took part in a focus group (n=7, consisting of between 2 and 4 participants) or an individual interview (n=4; Multimedia Appendix 3).

A total of 11 pregnant or recently pregnant women took part in think-aloud interviews, and 10 pregnant women tested the DAPHNY app at home. Participant characteristics are displayed in Multimedia Appendix 3.

### Findings

#### Health Care Professional Focus Groups and Early Feasibility Testing, Think-Aloud Interviews

**Figure 3.**
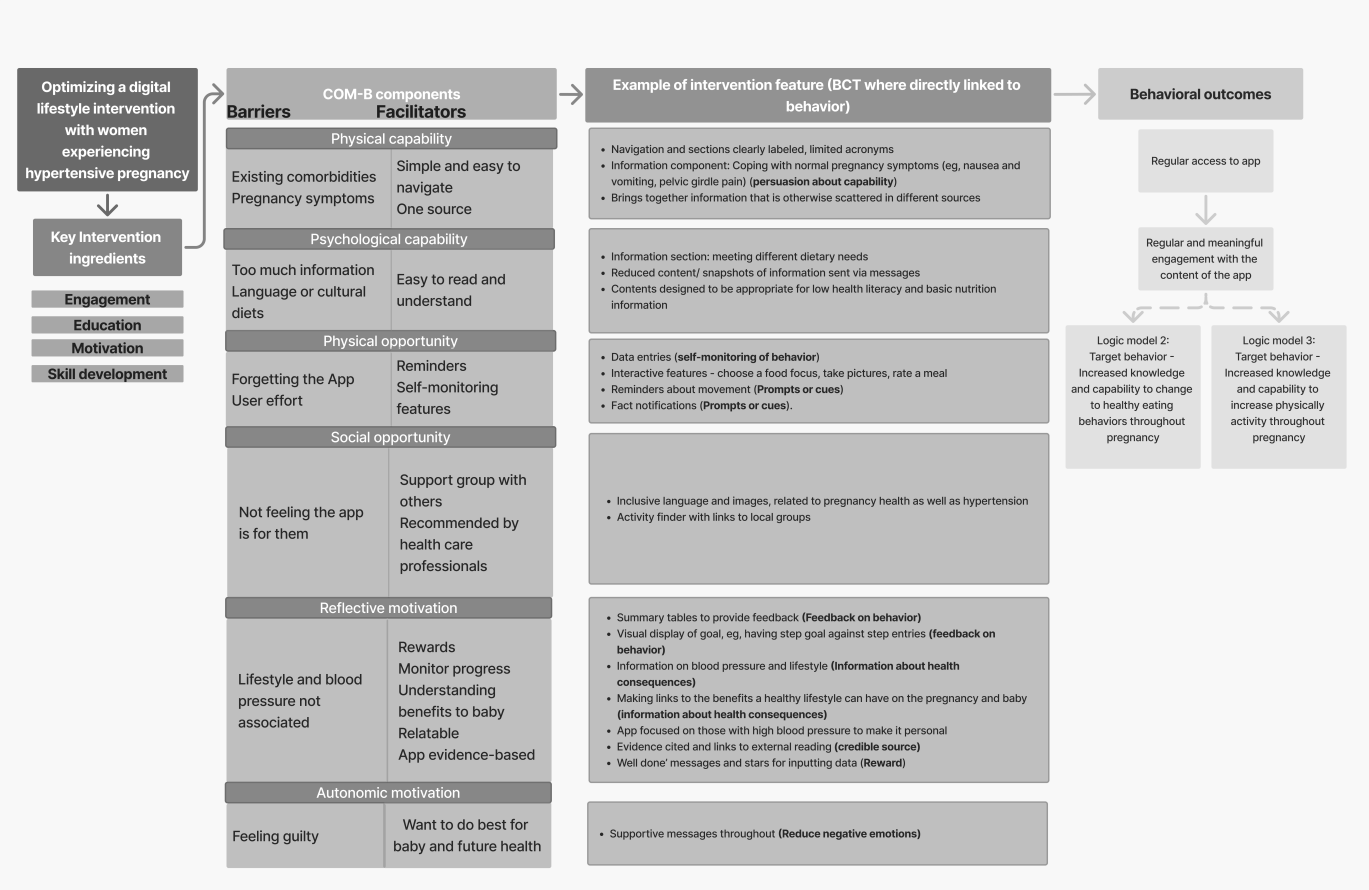
Logic model 1: target behavior—accessing and engaging with a lifestyle app. BCT: behavior change technique; COM-B: capability opportunity motivation-behavior model.

**Figure 4.**
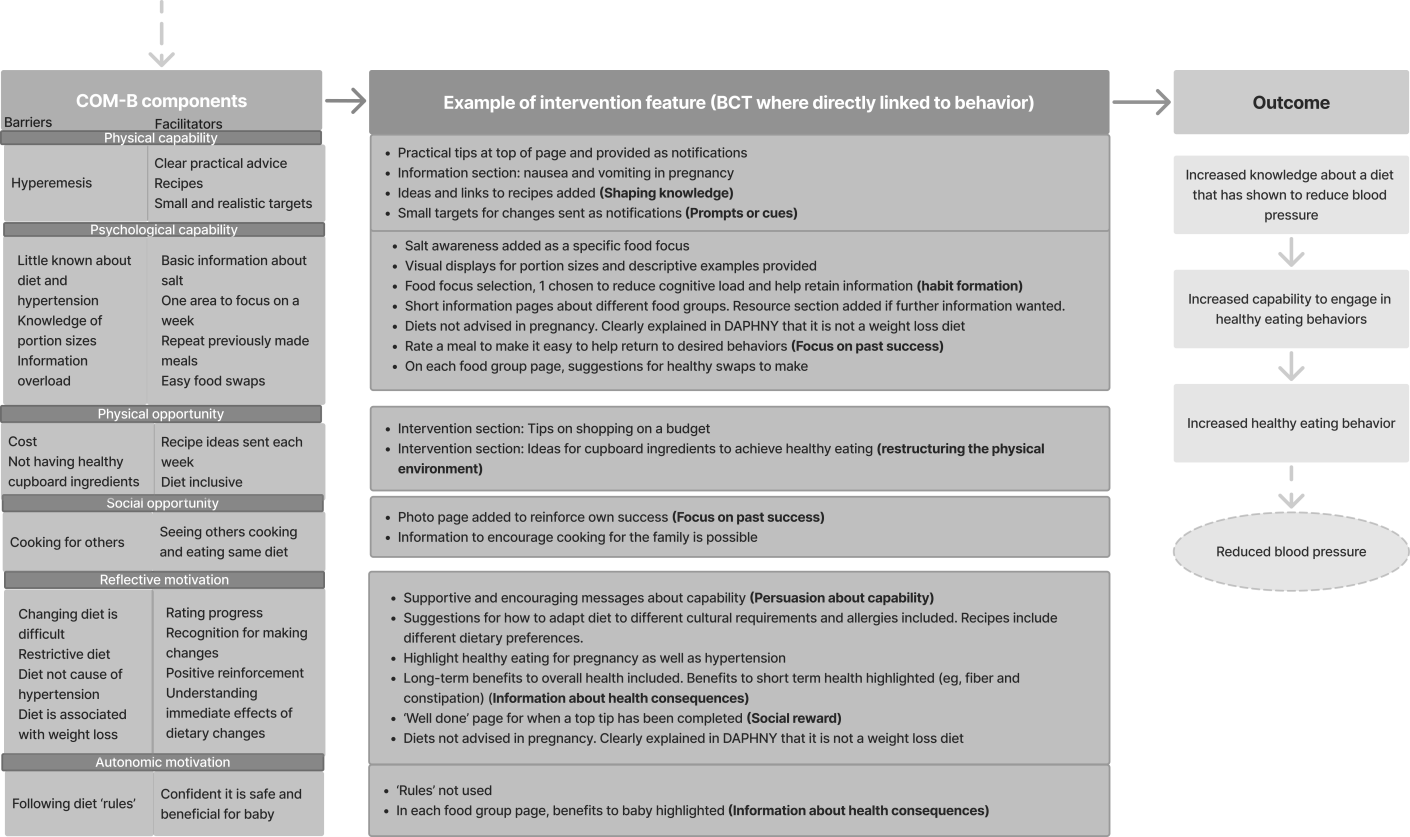
Logic model 2: target behavior—increased knowledge and capability to change to healthy eating behaviors throughout pregnancy. BCT: behavior change technique; COM-B: capability opportunity motivation-behavior model.

**Figure 5.**
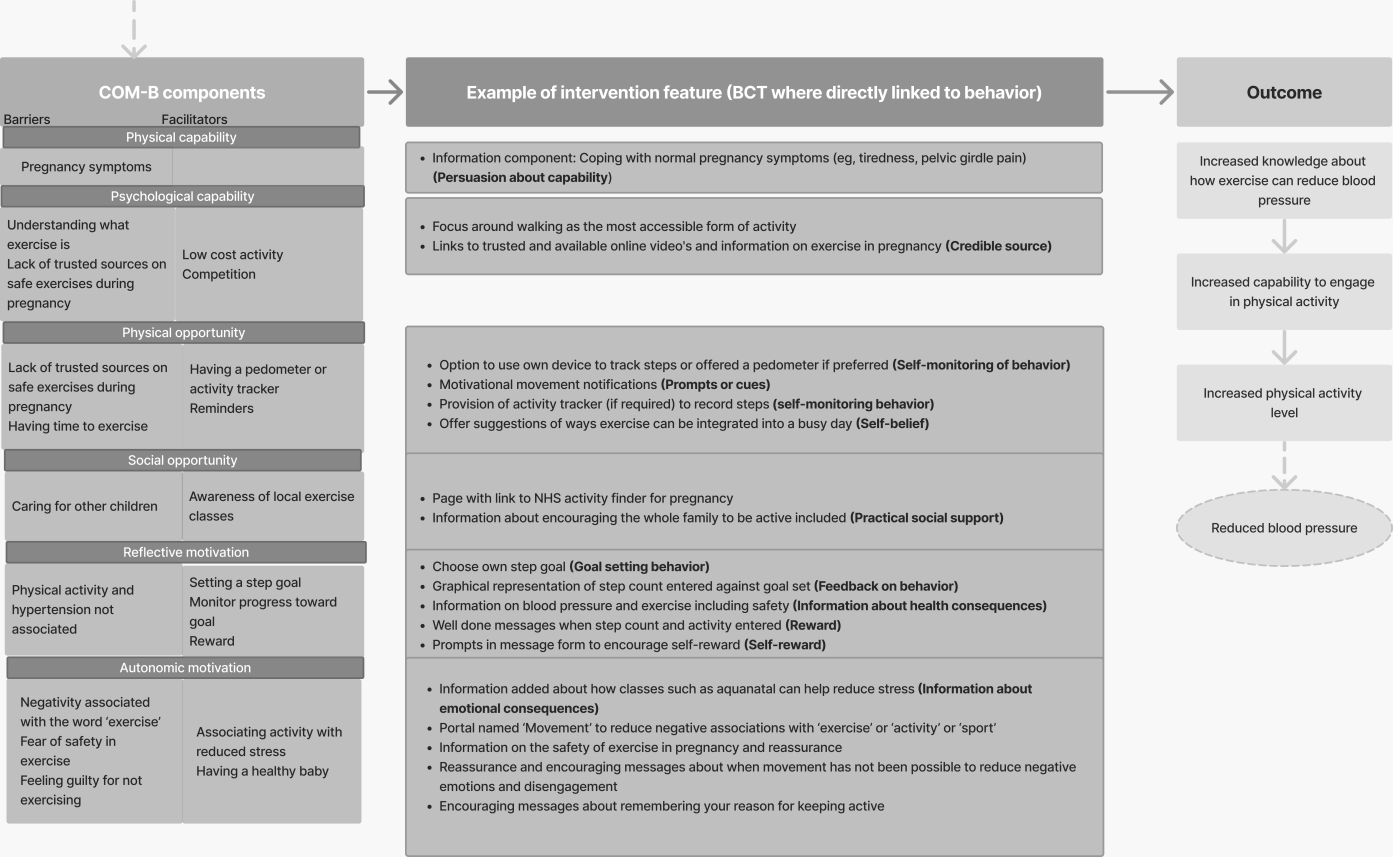
Logic model 3: target behavior: Increased knowledge and capability to increase physical activity throughout pregnancy. BCT: behavior change technique; COM-B: capability opportunity motivation-behavior model.

The table of changes informed the production of three logic models. Logic model 1 (Figure 3) acts as a mediating process that is necessary to lead to logic models 2 (Figure 4) and 3 (Figure 5); that is, accessing and engaging with the app is required to increase knowledge and capability to increase healthy eating and physical activity levels during pregnancy. Subsequent changes in blood pressure are outcomes associated with logic models 2 and 3, but were not a testable part of the intervention or within the scope of this study.

Table 1 displays an abridged excerpt from the table of changes to demonstrate an example of participant views and changes implemented.

**Table 1.** Abridged excerpt from the table of changes that informed changes to the intervention and logic models.

App page	Blood pressure–focused foods	Blood pressure portal	Blood pressure and your lifestyle	Movement portal	Weight portal	Reflections or free writing page
Participant comments	A. “really break things down into achievable steps ... they can seem overwhelming altogether ... like 1 little thing to think about” B. “it’s quite information intense, isn’t it?...”	“...include some information that what will be the effects if blood pressure is not controlled during pregnancy”	A. “I feel like if you have loads of information some people might read it all, but you might need it punchier with the option to click on it and read more. Need easy and quick to absorb information.” B. “you don’t want to be accusatory do you, I think that can be, people shut off immediately” “it would have probably ended up in another layer of guilt over stuff I wasn’t doing that I knew I should have been doing”	A. “Even if it was a slow paced swim and then it was just kind of, told me Great, you’ve done something today, well done. I’d quite like that.” B. “does the app have a pedometer built into it because that would be good”	“I would do it but I think ... I would be probably a bit upset to see it on a graph not showing where I want it to be. This would demotivate me.”	“I like tracking my blood pressure. That data I put into an app because I get something from it, which is an important monitoring of an important metric, but kind of moods and even symptoms when it’s not looking for a particular pattern, though, I just I just wouldn’t bother.”
Decision for change^a^	A. Said repeatedly, Important for behavior change. B. Important for behavior change	Experience, Important for behavior change	A. Important for behavior change, easy and uncontroversial. B. Supported by experience, said repeatedly, important for behavior change	A. Experience, important for behavior change B. Not changed	Experience	Said repeatedly, experience, does not contradict
MoScoW^b^ criteria^c^	A. Should have B. Must have	Must have	A. Should have B. Must have	A. Should have B. Would like	Should have	Could have
Action taken	A. App presents a list of top tips which participants can choose to receive as a notification. Emphasis on how to break goals down into small steps. B. Simplified language and reduced text. Links to further information if wanted by some.	Short- and long-term effects of high blood pressure for both mother and baby made clear.	A. Links to more information for those who wish to read more. B. Tone and terms used throughout app supportive. Section to acknowledge difficulties to avoid adding additional burden or feelings of guilt.	A. “Well done” message prompted when an activity is entered. B. Not feasible but important consideration for future iterations.	Information about weight not included on main homepage. Made an optional section, women can choose to engage with this or not.	Page removed as unlikely to impact target behaviour therefore its removal does not contradict guiding principles.

a See Multimedia Appendix 2 for coding framework definitions.

b MoSCOW: Must have, Should have, Could have, Would like.

c Bradbury et al [37].

Key features included supportive messaging, acknowledging the challenges of being pregnant, goal setting, rewards and recognition that effort has been made, feedback on behavior including self-monitoring data, prompts via push notifications, shaping knowledge around how to have healthy behaviors, information about health consequences such as pregnancy outcomes associated with poor blood pressure control, and the information on the app being from a credible source and therefore sources used clearly referenced.

**Figure 6.**
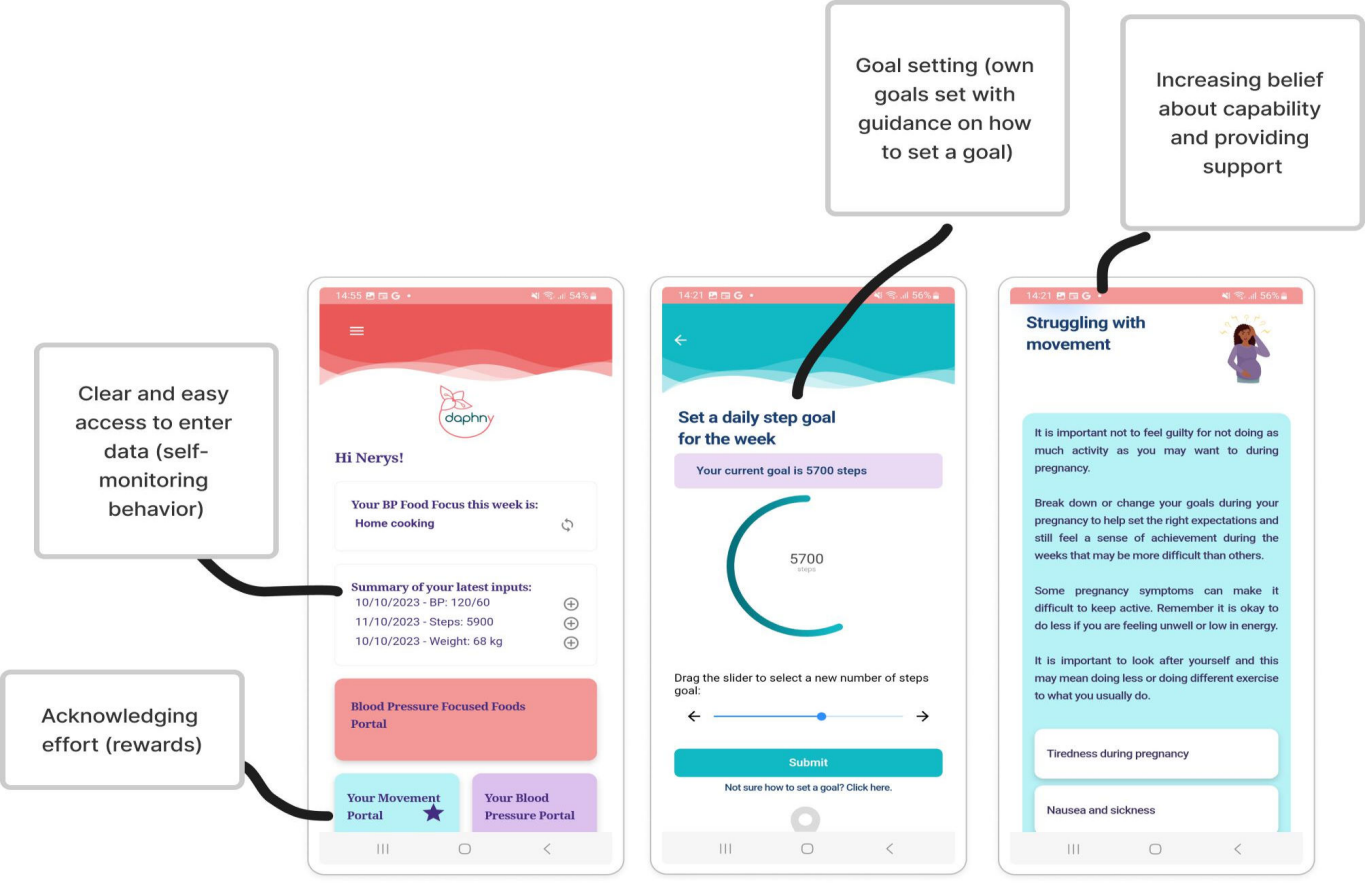
Screenshots of the DAPHNY (Diet and Activity for Pregnancy Hypertension) app prototype with associated behavior change techniques following iterative feedback from participant interviews and focus groups.

Figure 6 displays example screenshots with associated BCTs. Further screenshots can be viewed in Multimedia Appendix 4.

Some desirable features were not feasible within this intervention development study but are important findings to inform future iterations, supporting access, engagement, and implementation of the intervention (Table 2).

**Table 2.** Findings indicating important considerations for future iterations.

COM-B^a^ component	Findings	Future features to consider
Physical capability	Not all women have smartphones or use apps Downloadable on different devices Clear meal plans	Printable or web version Weekly meal plans with shopping list
Psychological capability	Only available in English	Available in different languages
Physical opportunity	Health care professionals do not have training on lifestyle factors and hypertension Tool to help facilitate health care professionals talk about the app Linked devices Entering food intake to learn about intake Being able to measure portion sizes	Specific training for health care professionals about lifestyle factors and hypertension Implement a prompt for consultations to remind professionals to introduce the app Link to other devices and apps (relevant to self-monitoring features) Optional food tracking feature Specific lifestyle-related training Provision of food scales
Social opportunity	Recommended by a health care professional Support community Seeing other people’s stories Competition with others (steps)	Chat forum with other users or similar Success stories about making lifestyle changes Gamification with other users
Reflective motivation	Using the app in the postnatal or preconception period	Talk to GPs^b^ and other health care professionals about the app Consider application across the life course
Autonomic motivation	None identified	None identified

a COM-B: capability opportunity motivation-behavior.

b GP: general practitioner.

Following changes derived from focus groups and think-aloud interviews, Version 0.0.1 of the DAPHNY app was built. The app was tested for usability and functionality within the research team, including PPI contributors. Technical issues were resolved prior to user testing.

#### Early Feasibility Testing, User Testing

##### Summary of Total App Use

**Figure 7.**
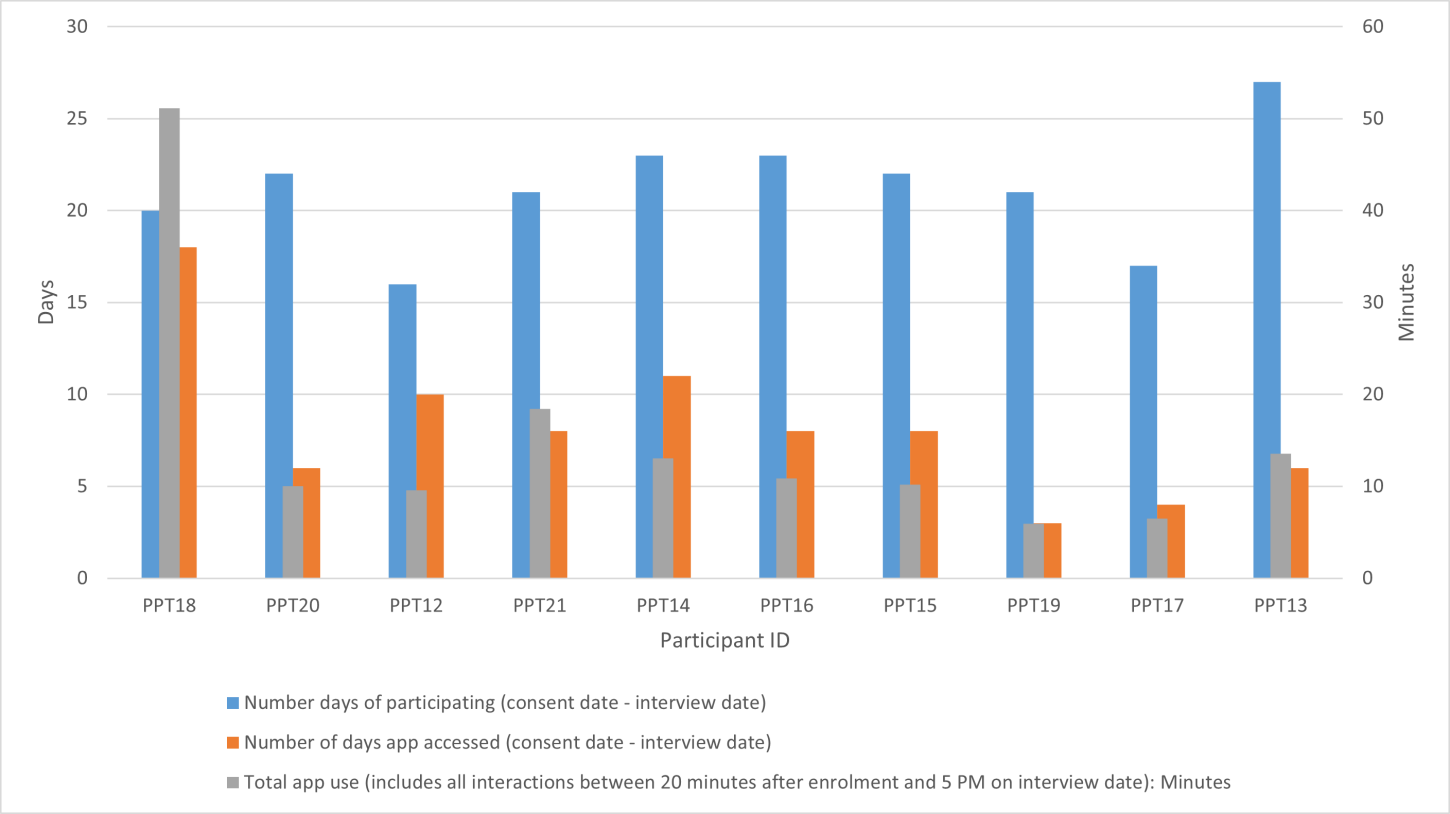
Total app use by each participant (ordered by no, sessions, high to low) during user testing.

Participants had access to the app for between 16 and 27 days. The proportion of days participants accessed the app within this time varied from 14% to 90%. The total number of minutes a participant accessed the app over the course of their participation ranged from 5 minutes 57 seconds (PPT19) to 51 minutes 8 seconds (PPT18; Figure 7).

PPT18 spent the most time, had the greatest number of sessions (23) on the app, and accessed it on the majority of days during participation (90%).

Spending fewer days on the app did not necessarily equate to less time on the app. For example, PPT20 and PPT12 spent a similar amount of time on the app (10 min 1 s and 9 min 34 s, respectively), yet for PPT12, this was spread over 4 more days than PPT20, indicating more frequent access to the app but shorter sessions.

There were 116 sessions in total (Table 3). The majority of the sessions had durations that were more akin to the minimum duration, with a median length of 36 seconds.

**Table 3.** Summary of app sessions during the user-testing period.

	Mean (SD)	Median	Range (min-max)
Number of sessions per participant	13 (6.84)	13	3‐23
Duration of sessions (mm:ss)^a^	01:19 (02:27)	00:36	00:05-05:20

a A 21:40 minute session was removed as only one page (step summary page) was accessed during this time, suggesting the app was left open but with no active interaction from the participant. Mean and median are calculated from the inclusion of 21:40.

##### Pages Accessed

The number of individual pages that the participants accessed ranged from 7 to 23 pages. The pages requiring the user to take an action, such as blood pressure readings and choosing a food focus, were accessed by more participants than the pages with written information, which required a deeper level of engagement through multiple layers of the app. Data entry pages “Enter a clinic blood pressure reading” (40 sessions) and “Record today’s step count” (39 sessions) had the greatest number of sessions, indicating that participants’ use of the app was predominantly action-based as opposed to reading information.

The least explored area of the app was the food portal. Despite this finding, participants spoke about how it was useful having this information in one place where they could find out more if they needed it. Furthermore, information about food was received in notifications, which may have led to reduced interaction with this section.

The blood pressure portal was the most frequently accessed portal (24 sessions). The “Explore more” portal had 3 sessions. This portal included information on weight and healthy living after giving birth, as well as stress, sleep, alcohol, and smoking information, indicating that few participants accessed these additional subsections, contradicting a desire for these in think-aloud interviews.

##### Entering Data

Although participants were instructed to only enter blood pressure readings from the clinic, four participants entered blood pressure readings between 4 and 19 submissions, suggesting some were using it to record their self-monitoring readings. There was a keenness to have some of the information available on the DAPHNY app in the same place as they were entering their self-monitoring blood pressure reading.

Five participants set a step goal, 3 of whom did this during the enrollment session. In total, 6 participants entered a step count between 2 and 12 times, of whom 2 of these 6 did not set a step goal. Some participants did not enter a step count because they recorded this elsewhere on apps linked to existing activity trackers or their phone, indicating a need for interconnectivity between devices.

Few participants used the weight tracking feature. The weight section required navigation through multiple pages of the app, and therefore, some participants were not aware it existed. In some cases, participants did not feel this was needed as there was enough to explore in the movement, diet, and blood pressure sections. The focus on nutrients, as opposed to weight, was more acceptable to some participants.

## Discussion

### Summary of Findings

This study led to the development of an evidence-based, theoretically informed mobile lifestyle app (the DAPHNY app) that, through methods of the PBA, prioritized the needs and perspectives of those with chronic hypertension during pregnancy. Participant feedback informed three logic models that identified key intervention features to optimize the intervention. Multiple iterations of the app implementing these changes led to a first version that was user tested by a group of women. Action-based features were accessed more frequently than educational pages. Strategies to increase engagement, improve acceptability, and support implementation were offered by participants.

### Comparison to the Wider Literature

Women in the study were accustomed to self-monitoring their blood pressure, and some of the information on the app was considered an acceptable addition to existing practices. However, engagement in the lifestyle information was relatively limited, considering the extent of available content. A study that developed an intervention for nonpregnant patients with hypertension found that the lifestyle component had little traction with participants, especially if medication sufficiently controlled blood pressure, suggesting a lack of overall engagement in this part of blood pressure management [27]. This has also been demonstrated in a systematic review, which found that promoting positive lifestyle behaviors within hypertensive populations was met with a lack of willingness, even where there was knowledge of the benefits [39]. Embedding lifestyle-promoting messages effectively that lead to meaningful change within hypertensive populations remains a challenge.

Barriers to engaging with healthy behaviors that were identified by women with chronic hypertension in the study were similar to those identified by the general pregnant population. These included common pregnancy symptoms such as general discomfort, sickness or tiredness, and work or family commitments, as well as safety concerns [40-42]. Furthermore, cost and time have been found by others to be major barriers in consuming heart-healthy diets among women with risk factors for hypertension [43]. The DAPHNY app addressed some of these barriers through codeveloping supportive, practical, and realistic messaging to manage cognitive load and reduce feelings of guilt. Sharing goals, progress, and efforts with a health care professional, who women view as a credible source of information and support, alongside the app, was important to mitigate some concerns shared by participants in the study. This aligns with research that developed an activity app for women with gestational diabetes [44]. Health care professionals’ health promotion role and how they deliver this information must be examined to understand if the app could be used as a tool to facilitate this partnership with women.

Women were keen to be acknowledged for their efforts through a reward and recognition system via the app. This may reflect women’s desire to be recognized for their work as expectant mothers whilst navigating a system that is oriented around monitoring and medication regimes. Bringing women back into the conversation about their own bodies, including ways they may manage blood pressure in their personal domain, beyond the maternity care setting, could contribute toward opening up the dialogue where women can believe in the capabilities of their own bodies. The DAPHNY app gives women a tool by providing content that is woman-centered, the underlying philosophy of the app, to acknowledge the experiences through hypertensive pregnancy, its challenges, and provide support to manage or overcome these.

The notorious challenge of multiple, already available systems and their integration was brought to light in developing this digital intervention. For example, some women did not use the DAPHNY app to monitor their steps, because this was recorded elsewhere and automatically. Similar to others, health care professional participants expressed enthusiasm for the app to be linked to existing apps and electronic health records [45]. This supports the NHS England Better Births report and the NHS Long Term plan that value the role of digital tools in maternity care [17,46]. Though there is a significant preference across all participants for embedding and linking existing technologies, the complex landscape of both the digital field and large health care systems continues to be a barrier to the seamless implementation of apps for the benefit of the end user [47]. Synchronizing a digital intervention across different trusts and professionals would require funding, national-level stakeholder involvement, and a host organization to manage, update, and provide governance to oversee the safety and ongoing usability of an intervention. Collaboration with those driving the digital agenda within maternity care is essential to bolster the development of a lifestyle intervention beyond its own silo. As a small first step toward interconnected systems, adjoining the findings from this development study with apps that are used within current care would be appropriate to investigate. This would benefit from embedding implementation theory to address barriers, prior to proceeding to a larger-scale feasibility trial to validate and more comprehensively understand if and how a digital lifestyle intervention for hypertensive pregnancy can be embedded within the NHS existing system and women’s lives.

### Limitations

The DAPHNY app was designed with experts and relevant users of the intervention, with continuous input from PPI representatives. It builds on a strong body of evidence that women can and are willing to self-manage their hypertension at home. Therefore, this study appropriately addresses further self-management strategies that may influence blood pressure management through pregnancy that have not been explored before [24,48,49].

The perspectives of those with long-term hypertension in relation to their lifestyle behaviors were elicited, which, to our knowledge, has not been done before. Pregnant women whose first language was not English were included, as well as those who followed their own cultural diets, which gave important insight when discussing the food-related sections of the app. There was limited diversity in the sample of participants in the think-aloud interviews in relation to ethnicity. However, 6 participants enrolled in user testing were from different non-White British ethnic backgrounds, thereby increasing the diversity of views heard compared to the first interviews. Furthermore, there was a range of educational attainment, which is a strength of the study, as those with lower education levels are underrepresented in research [50]. An important next step is to speak to women attending different NHS Trusts with diverse backgrounds to further learn how the provision of lifestyle information within the context of hypertension management may be adapted and optimized. Digital interventions may not address needs across populations, for example, those with low digital health literacy, limited internet access, or infrequent device use. However, insights from this study can inform the design of different models of information delivery to support health promotion that better meet the needs of women with chronic hypertension. The UK context of the study means findings may not translate across different economic and health care landscapes.

### Conclusions

The PBA led to the development of a novel, theoretically underpinned, evidence-based lifestyle app designed to address the needs and perspectives of women with chronic hypertension during pregnancy. Valuable insight from this important population provides new data that adjoins lifestyle behavior and hypertension during pregnancy that has previously been overlooked but has the potential to improve outcomes, as evidenced outside of pregnancy.

Future work must address the recommended changes and the learnings from this work with a view to moving toward the implementation and evaluation stage of the PBA so it can be formally piloted and tested for effectiveness on behavior change and subsequent blood pressure control. Good lifestyle support alongside regular monitoring and management of hypertension has the potential to improve cardiovascular health during pregnancy and beyond. This study is a step toward putting this on the public health and maternity agenda, which should be noted by professionals and researchers.

## Supplementary material

10.2196/68927 Multimedia Appendix 1 Planning phase of the person-based approach—overview of scoping the relevant literature for intervention development to inform prototypes.

10.2196/68927 Multimedia Appendix 2 Coding framework used to facilitate decisions about intervention modifications.

10.2196/68927 Multimedia Appendix 3 Participant characteristics for the health care professional focus groups (n=23), think-aloud interviews (n=11), and user testing (n=10).

10.2196/68927 Multimedia Appendix 4 Screenshots of key behavior change techniques within the intervention.

10.2196/68927 Checklist 1 GUIDED (Guidance for the Reporting of Intervention Development) checklist.

## Abbreviations

BCT: behavior change technique
COM-B: capability opportunity motivation-behavior model
DAPHNY: Diet and Activity for Pregnancy Hypertension
GUIDED: Guidance for the Reporting of Intervention Development
MoSCOW: Must have, Should have, Could have, Would like
NHS: National Health Service
PBA: person-based approach
PPI: patient and public involvement
