# Supplementary material for: A Digital Lifestyle App for Hypertension During Pregnancy: Mixed Methods Intervention Development Study Using the Person-Based Approach
Source: JMIR Form Res. 2025 Jul 18;9:e68927. doi: 10.2196/68927 (PMC12296244; doi:10.2196/68927)
Supplement: Multimedia Appendix 1 [file formative-v9-e68927-s001.docx]

| What evidence exists around lifestyle interventions for this population? | A systematic review examined the literature for lifestyle interventions for those entering pregnancy with existing hypertension and/ or diabetes [1].  Few studies examined the effect of lifestyle interventions in this population. |
| --- | --- |
| What is the role of lifestyle in hypertension? | The association between a healthy lifestyle and hypertension status is well established with poor diets and sedentary behaviour being associated with an elevated hypertension risk [2, 3].  Due to this, National guidance recommends hypertensive patients are provided with information about a healthy diet and exercise, amongst other health behaviour changes [4].  More specifically, the DASH (Dietary Approaches to Stop Hypertension) has been the most effective diet in creating meaningful reductions in blood pressure, with some identifying similar reductions as that produced by some medication regimes [5-7] |
| What lifestyle advice is given to pregnant women who have hypertension? | National guidance recommends pregnant women with hypertension are provided with information on managing weight, healthy eating and exercise in line with guidelines for the general hypertensive population.  However, healthcare professionals report both personal and contextual barriers in talking to pregnant women about health promoting behaviours [8-10].  As a result, women report receiving inconsistent advice, lack of support and confusion over the information they do receive [11-16].  There is a lack of effective tools to support healthcare professionals deliver this information in a collaborative and supportive manner that sufficiently equips women to make decisions about their lifestyle choices during pregnancy [17]. |
| Why a digital intervention during pregnancy? | Few available pregnancy Apps are based on robust scientific evidence, incorporation of effective behaviour change techniques and regulation of content and screening for co-morbidities [18-20].  Work within the wider research team has developed theoretically underpinned Apps for gestational diabetes and hypertension to self-monitor blood sugar levels and blood pressure control through pregnancy [21-25].  This expertise within the research group positions the DAPHNY study well to explore how lifestyle support can be delivered within an App, which is often received by women outside of the clinical context but may have important implications for blood pressure management. |

1. Goddard L, Patel R, Astbury NM, Tucker K, McManus RJ. Evidence of lifestyle interventions in a pregnant population with chronic hypertension and/or pre-existing diabetes: A systematic review and narrative synthesis. Pregnancy Hypertension. 2023;31:60-72. doi: 10.1016/j.preghy.2022.12.004.

2. Valenzuela PL, Carrera-Bastos P, Gálvez BG, Ruiz-Hurtado G, Ordovas JM, Ruilope LM, et al. Lifestyle interventions for the prevention and treatment of hypertension. Nature Reviews Cardiology. 2020. doi: 10.1038/s41569-020-00437-9.

3. Dickinson HO, Mason JM, Nicolson DJ, Campbell F, Beyer FR, Cook JV, et al. Lifestyle interventions to reduce raised blood pressure: a systematic review of randomized controlled trials. Journal of Hypertension. 2006;24(2).

4. National Institute for Health and Care Excellence (NICE). NG136: Hypertension in adults: diagnosis and management. 2019 [cited 2021 January]; Available from: https://www.nice.org.uk/guidance/ng136.

5. Ozemek C, Laddu DR, Arena R, Lavie CJ. The role of diet for prevention and management of hypertension. Current Opinion in Cardiology. 2018;33(4).

6. Filippou CD, Tsioufis CP, Thomopoulos CG, Mihas CC, Dimitriadis KS, Sotiropoulou LI, et al. Dietary Approaches to Stop Hypertension (DASH) Diet and Blood Pressure Reduction in Adults with and without Hypertension: A Systematic Review and Meta-Analysis of Randomized Controlled Trials. Advances in Nutrition. 2020;11(5):1150-60. doi: 10.1093/advances/nmaa041.

7. Gay HC, Rao SG, Vaccarino V, Ali MK. Effects of different dietary interventions on blood pressure: systematic review and meta-analysis of randomized controlled trials. Hypertension. 2016;67(4):733-9.

8. Heslehurst N, Newham J, Maniatopoulos G, Fleetwood C, Robalino S, Rankin J. Implementation of pregnancy weight management and obesity guidelines: a meta-synthesis of healthcare professionals' barriers and facilitators using the Theoretical Domains Framework. Obesity Reviews. 2014;15(6):462-86. doi: 10.1111/obr.12160.

9. Heslehurst N, Russell S, McCormack S, Sedgewick G, Bell R, Rankin J. Midwives perspectives of their training and education requirements in maternal obesity: A qualitative study. Midwifery. 2013;29(7):736-44. doi: 10.1016/j.midw.2012.07.007.

10. Atkinson L, French DP, Ménage D, Olander EK. Midwives' experiences of referring obese women to either a community or home-based antenatal weight management service: Implications for service providers and midwifery practice. Midwifery. 2017;49:102-9. doi: 10.1016/j.midw.2016.10.006.

11. Christenson A, Johansson E, Reynisdottir S, Torgerson J, Hemmingsson E. Shame and avoidance as barriers in midwives’ communication about body weight with pregnant women: A qualitative interview study. Midwifery. 2018;63:1-7. doi: 10.1016/j.midw.2018.04.020.

12. Christenson A, Johansson E, Reynisdottir S, Torgerson J, Hemmingsson E. “… or else I close my ears” How women with obesity want to be approached and treated regarding gestational weight management: A qualitative interview study. PLoS One. 2019;14(9):e0222543.

13. Smith D, Lavender T. The maternity experience for women with a body mass index ≥ 30 kg/m2: a meta-synthesis. BJOG: An International Journal of Obstetrics & Gynaecology. 2011;118(7):779-89. doi: 10.1111/j.1471-0528.2011.02924.x.

14. Nikolopoulos H, Mayan M, MacIsaac J, Miller T, Bell RC. Women’s perceptions of discussions about gestational weight gain with health care providers during pregnancy and postpartum: a qualitative study. BMC Pregnancy and Childbirth. 2017;17(1):97. doi: 10.1186/s12884-017-1257-0.

15. Brown A, Avery A. Healthy weight management during pregnancy: what advice and information is being provided. Journal of Human Nutrition and Dietetics. 2012;25(4):378-87. doi: 10.1111/j.1365-277X.2012.01231.x.

16. Lavender T, Smith DM. Seeing it through their eyes: a qualitative study of the pregnancy experiences of women with a body mass index of 30 or more. Health Expectations. 2016;19(2):222-33. doi: 10.1111/hex.12339.

17. Goddard L, Astbury NM, McManus RJ, Tucker K, MacLellan J. Clinical guidelines for the management of weight during pregnancy: a qualitative evidence synthesis of practice recommendations across NHS Trusts in England. BMC Pregnancy and Childbirth. 2023;23(1):164. doi: 10.1186/s12884-023-05343-9.

18. Hayman M, Alfrey K-L, Cannon S, Alley S, Rebar AL, Williams S, et al. Quality, Features, and Presence of Behavior Change Techniques in Mobile Apps Designed to Improve Physical Activity in Pregnant Women: Systematic Search and Content Analysis. JMIR Mhealth Uhealth. 2021;9(4):e23649. doi: 10.2196/23649.

19. Musgrave LM, Kizirian NV, Homer CSE, Gordon A. Mobile Phone Apps in Australia for Improving Pregnancy Outcomes: Systematic Search on App Stores. JMIR Mhealth Uhealth. 2020;8(11):e22340. doi: 10.2196/22340.

20. Hayman M, Alfrey K, Waters K, Cannon S, Mielke G, Keating S, et al. How APPropriate are physical activity apps for pregnant women appropriate: A systematic search and content analysis of evidence-based content, features of exercise instruction, and expert involvement. JMIR Preprints: 27/06/2021:31607. 2021.

21. Smith R, Michalopoulou M, Reid H, Riches SP, Wango YN, Kenworthy Y, et al. Applying the behaviour change wheel to develop a smartphone application ‘stay-active’ to increase physical activity in women with gestational diabetes. BMC Pregnancy and Childbirth. 2022;22(1):253. doi: 10.1186/s12884-022-04539-9.

22. Band R, Hinton L, Tucker KL, Chappell LC, Crawford C, Franssen M, et al. Intervention planning and modification of the BUMP intervention: a digital intervention for the early detection of raised blood pressure in pregnancy. Pilot and Feasibility Studies. 2019;5(1):153. doi: 10.1186/s40814-019-0537-z.

23. Mackillop L, Loerup L, Bartlett K, Farmer A, Gibson OJ, Hirst JE, et al. Development of a Real-Time Smartphone Solution for the Management of Women With or at High Risk of Gestational Diabetes. Journal of Diabetes Science and Technology. 2014;8(6):1105-14. doi: 10.1177/1932296814542271.

24. Hirst JE, Mackillop L, Loerup L, Kevat DA, Bartlett K, Gibson O, et al. Acceptability and user satisfaction of a smartphone-based, interactive blood glucose management system in women with gestational diabetes mellitus. Journal of diabetes science and technology. 2015;9(1):111-5. PMID: 25361643. doi: 10.1177/1932296814556506.

25. Tucker K, Rose F, Lavallee L, Roman C, Goddard L, McManus RJ. Intervention development and optimisation of a multi-component digital intervention for the monitoring and management of hypertensive pregnancy: the My Pregnancy Care Intervention. Pilot and Feasibility Studies. 2024;10(1):139. doi: 10.1186/s40814-024-01562-9.
