# Supplementary material for: A Digital Lifestyle App for Hypertension During Pregnancy: Mixed Methods Intervention Development Study Using the Person-Based Approach
Source: JMIR Form Res. 2025 Jul 18;9:e68927. doi: 10.2196/68927 (PMC12296244; doi:10.2196/68927)
Supplement: Multimedia Appendix 2 [file formative-v9-e68927-s002.docx]

| **Code** | **Stands for** | **Means** |
| --- | --- | --- |
| **IMP** | Important for behaviour change | Likely to impact behaviour change or a precursor to behaviour change (e.g. acceptability, feasibility, persuasiveness, motivation, engagement) |
|  |  | Or is in line with the Logic Model |
|  |  | Or is in line with the Guiding Principles |
| **EAS** | Easy and uncontroversial | Easy change that doesn’t involve major design changes |
| **REP** | Repeatedly | Said repeatedly, by more than one participant |
| **EXP** | Experience | Something supported by the experience from Patient and Public Involvement |
|  |  | Evidence from the literature |
| **NCON** | Does not contradict | Does not contradict experience, Logic Model, or Guiding Principles |
| **NC** | Not changed | e.g. Not feasible |
|  |  | Or only one person said this |
