# Supplementary material for: A Digital Lifestyle App for Hypertension During Pregnancy: Mixed Methods Intervention Development Study Using the Person-Based Approach
Source: JMIR Form Res. 2025 Jul 18;9:e68927. doi: 10.2196/68927 (PMC12296244; doi:10.2196/68927)
Supplement: Multimedia Appendix 3 [file formative-v9-e68927-s003.docx]

Table S1. HCP Focus groups: Participant characteristics (n=23)

| **Profession** | |
| --- | --- |
| Midwife | 14 |
| Doctor | 5 |
| Student Midwife | 1 |
| Support/ assistant practitioner | 3 |
| **Main orientation** | |
| Rotational (more than one area of maternity) | 13 |
| Antenatal ward | 2 |
| Community and/ or MLU | 6 |
| Research | 1 |
| Other | 1 |
| **No. years’ experience** | |
| 0-5 | 7 |
| 6-10 | 4 |
| 11-15 | 6 |
| 16-20 | 4 |
| 20+ | 2 |

Table S2. Early Feasibility Testing, Think-aloud interviews - Participant characteristics (n=11)

| **Age category** | |
| --- | --- |
| 25-34 | 6 |
| 35-49 | 5 |
| **Current pregnancy status** | |
| Currently pregnant | 8 (Gestational age range 22+5/40 – 37+4/40) |
| Pregnant within last 12 months | 3 |
| **Gravidity** | |
| 1 | 6 |
| ≥2 | 5 |
| **Hypertension diagnosis** | |
| During pregnancy (<20/40) | 5 |
| Pre-pregnancy | 6 |
| **Ethnicity** | |
| White British | 8 |
| White European | 2 |
| Asian | 1 |
| **Highest education attainment** | |
| Other | 4 |
| Degree or above | 7 |

Table S3. Early feasibility testing, User Testing: Participant characteristics (n=10)

| **Age category** | |
| --- | --- |
| 18-24 | 2 |
| 25-34 | 4 |
| 35-49 | 4 |
| **Gravidity** | |
| 1 | 2 |
| ≥2 | 8 |
| **Hypertension diagnosis** | |
| During pregnancy (<20/40) | 5 |
| Pre-pregnancy | 5 |
| **Ethnicity** | |
| White British | 4 |
| White European and South African | 1 |
| South African | 1 |
| Asian | 1 |
| Black African | 2 |
| Arab | 1 |
| **Highest education attainment** | |
| Other | 6 |
| Degree or above | 4 |
