# Supplementary figures and images for: A Digital Lifestyle App for Hypertension During Pregnancy: Mixed Methods Intervention Development Study Using the Person-Based Approach
Source: JMIR Form Res. 2025 Jul 18;9:e68927. doi: 10.2196/68927 (PMC12296244; doi:10.2196/68927)

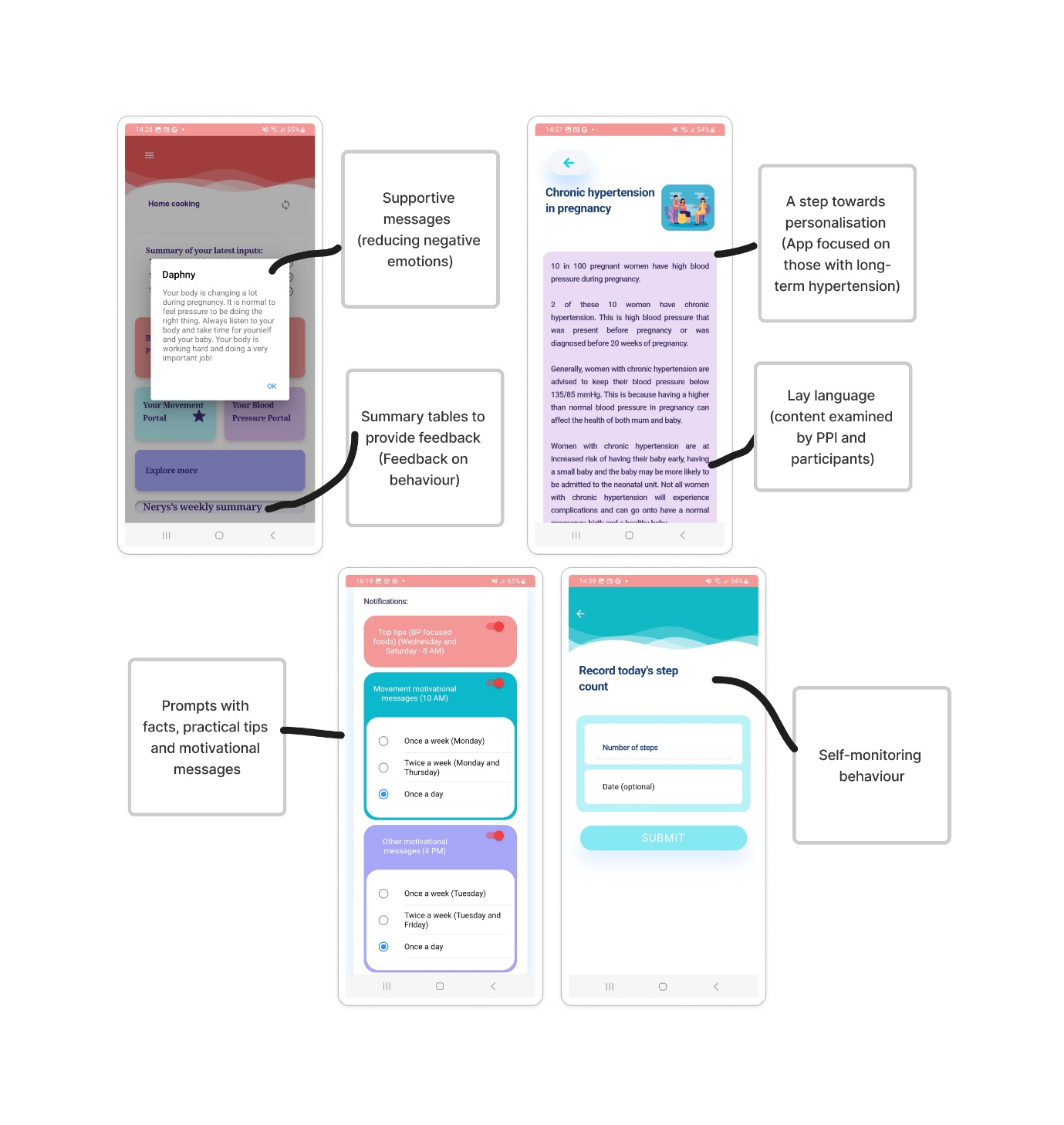

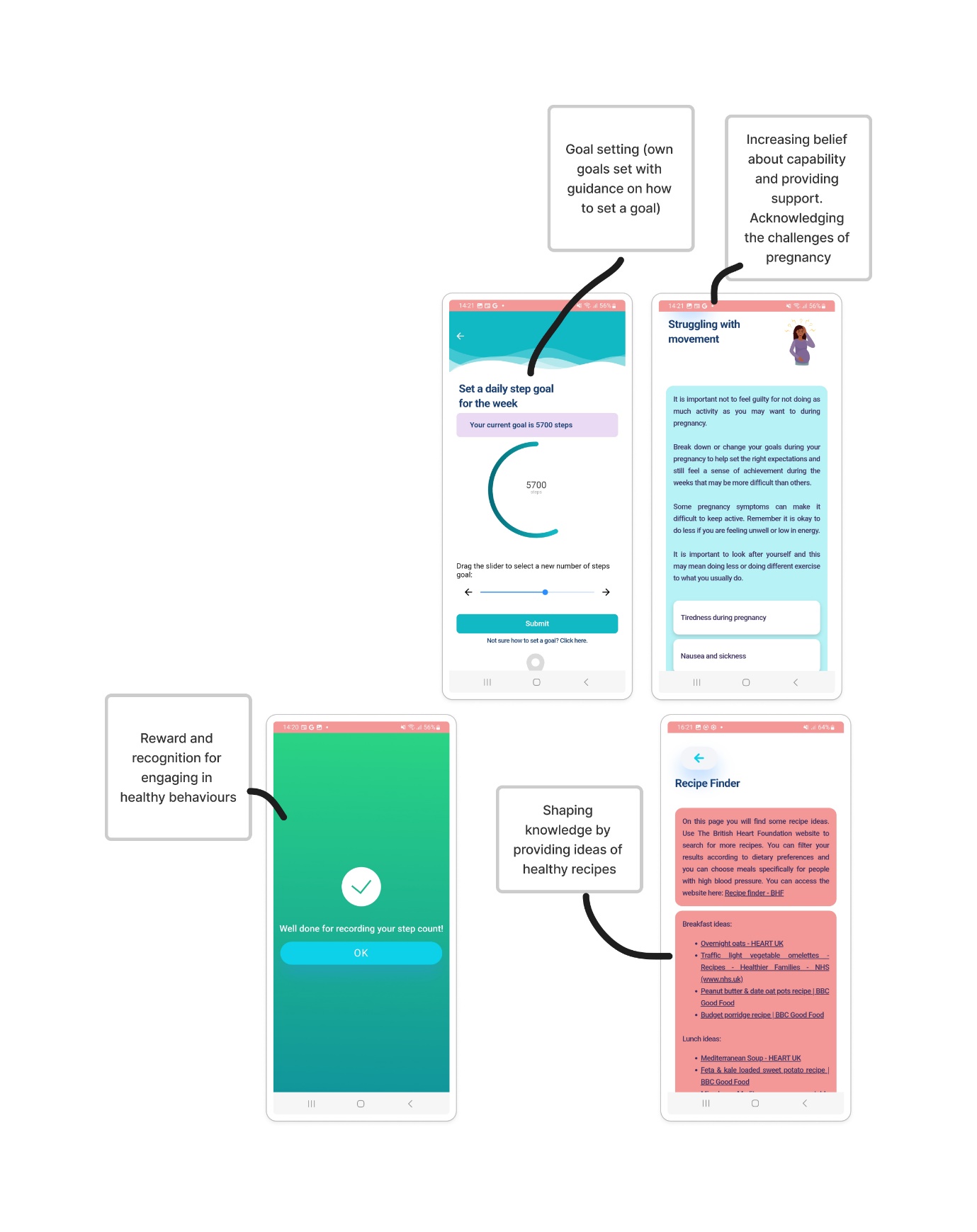

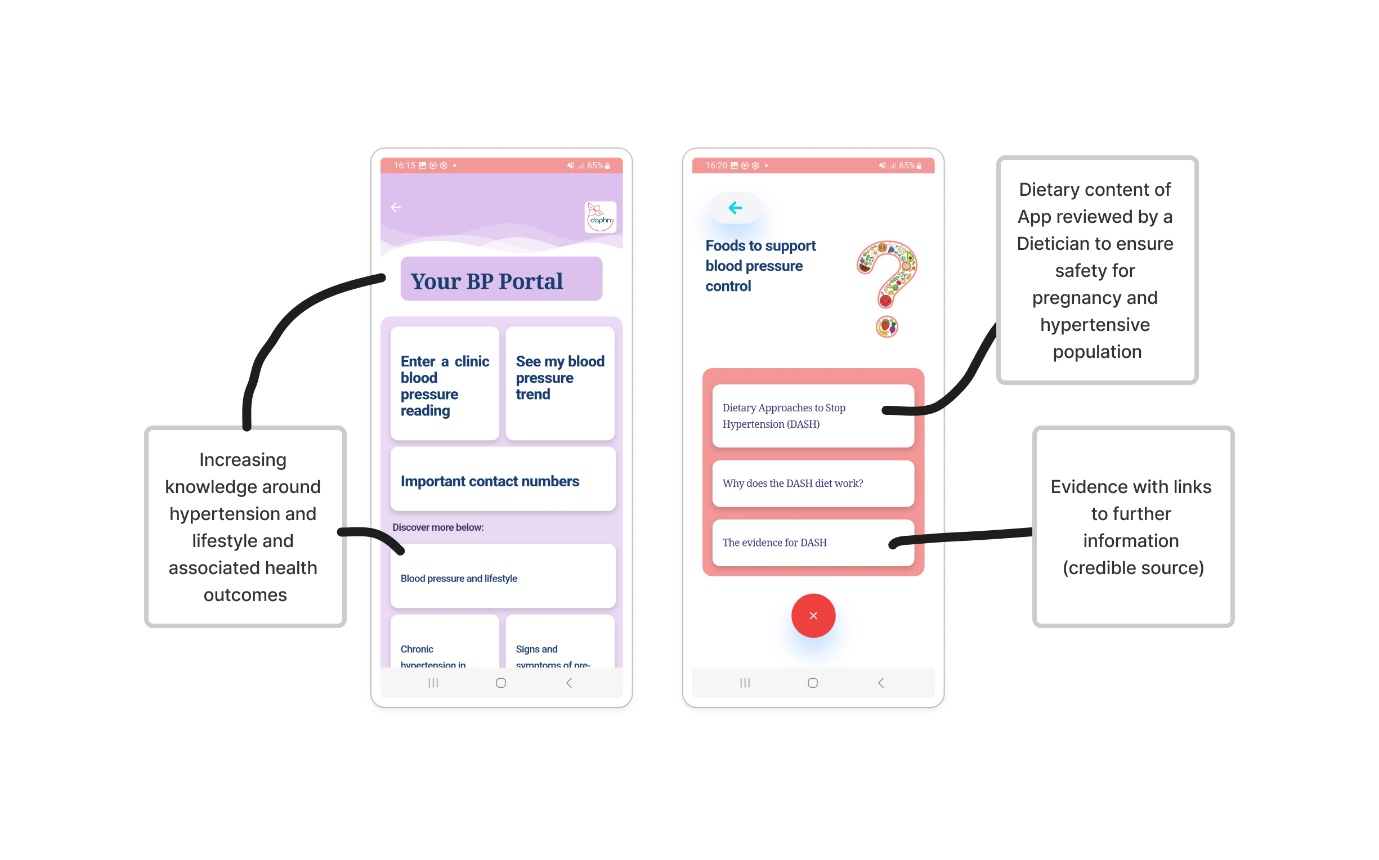

Supplement: Multimedia Appendix 4 [file formative-v9-e68927-s004.docx]
